# *Trichoderma harzianum* M2 Induces Systemic Resistance to Fusarium Crown Rot and Remodels Root Architecture to Promote Phosphorus Uptake in Wheat

**DOI:** 10.3390/plants15192957

**Published:** 2026-09-28

**Authors:** Junchang Li, Yingying Jin, Yingxue Wang, Yiying Chen, Tingyu Wang, Xingdong Yang

**Affiliations:** 1 Henan Province Key Laboratory of Efficient Crop Production and Food Quality Safety, Zhoukou Normal University, Zhoukou 466001, China; 15136258681@163.com; 2 Modern Agricultural Industry Research Institute, Henan Zhoukou Agricultural High-Tech Industry Demonstration Zone, Zhoukou 477100, China; 3 Wheat Technology Innovation Center, Henan Zhoukou Agricultural High-Tech Industry Demonstration Zone, Zhoukou 477100, China

**Keywords:** *Trichoderma*, wheat, FCR, induced systemic resistance, biocontrol

## Abstract

Wheat production is severely constrained by Fusarium crown rot (FCR) caused by *Fusarium pseudograminearum* and low soil nutrient use efficiency, making biocontrol an important strategy for sustainable agriculture. This study characterized a newly isolated *Trichoderma harzianum* strain, M2, and explored its bioprotective and growth-promoting mechanisms in wheat. Identified via morphological and phylogenetic analyses, strain M2 exhibited 91.24% in vitro inhibition against pathogenic *F. pseudograminearum*. Its 10^5^ cfu/mL spore suspension remodeled root architecture and increased seedling biomass in hydroponic and pot experiments. Greenhouse and field assays recorded FCR control efficacies of 90.12% and 71.70%, respectively; field application of M2 reduced the disease index from 82% to 28%. RNA-seq and qRT-PCR indicated that M2 confers FCR resistance by triggering induced systemic resistance. Microscopy demonstrated that M2 penetrates root epidermal cells, forms biofilm-like structures and stably colonizes root xylem tissues. Furthermore, M2 enhanced soil phosphorus uptake and phosphorus accumulation in wheat. Collectively, endophytic root colonization and multi-hormone regulatory pathways enable *T. harzianum* M2 to improve wheat defense and performance, representing a promising agent for eco-friendly wheat production.

## 1. Introduction

Wheat is one of the world’s major cereal crops, providing 20% of the annual energy supply [1]. The Food and Agriculture Organization of the United Nations (FAO) has set a target of increasing food production by 60% by 2050 [2]. To achieve this goal, the production of major food crops, including wheat, needs to continue to increase [3]. Wheat diseases are an important factor threatening the high yield, stable yield and high quality of wheat [4], including *Fusarium* head blight, powdery mildew and sharp eyespot, among which wheat Fusarium crown rot (FCR) poses severe threats [5]. FCR is a typical soil-borne disease caused by *Fusarium* strains. The main pathogens isolated from different regions include *F. pseudograminearum* (*Fp*), *F. culmorum* (*Fc*), *F. graminearum* (*Fg*), *F. oxysporum* (*Fo*) and *F. moniliforme* (*Fm*). Among these pathogens, *Fp* exhibits strong virulence, wide distribution and serious harm [6,7]. In addition, during the occurrence of FCR, it not only causes serious yield loss and economic loss, but also produces a variety of toxins and secondary metabolites, which cause serious harm to human and animal health [8]. Moreover, the pathogens of wheat FCR have a wide range of hosts and can survive for many years on soil stubble, and the disease site is at the base of the stem and the infection period is long, which makes control challenging [9].

*Trichoderma*, as a common soil fungus, has been shown to promote plant growth and is considered to be a group of biocontrol microorganisms with the most promising development prospects [10]. As a biocontrol fungus, it can colonize internal plant tissues, establish mutualistic symbiosis with host plants, and boost plant disease resistance alongside plant growth promotion [11]. Currently, *Trichoderma* spp. are widely applied in biocontrol, soil improvement, and plant growth promotion [12]. In 1997, it was proven for the first time that *Trichoderma* can induce ISR (induced systemic resistance) in plants [13]. ISR enables plants to mount rapid defensive responses upon pathogen attack [14]. ISR is phenotypically similar to SAR (systemic acquired resistance) caused by pathogen infection, but does not produce anaphylaxis in host plants.

It has been found that *T. virens*, *T. sperellum*, *T. atroviride*, *T. harzianum*, etc. can induce local or systemic resistance of plants to broad-spectrum pathogenic fungi, bacteria, viruses and other microorganisms [15]. For example, the expression of PR (pathogen-related) proteins was upregulated in leaves treated with *T. harzianum* and *Bacillus pumilus*, and the concentrations of peroxidase, endochitinase and β-1,3-glucanases were significantly increased and simultaneously enhanced wheat resistance to stem rot [16]. Similarly, it was found that metabolites derived from *T. harzianum* strain T-Soybean under field conditions could significantly inhibit the growth of pathogenic *Fusarium graminearum* (*Fg*), induce maize systemic resistance by increasing the activity of antioxidant enzymes, and increase corn yield [17]. Upon treatment of *Populus euphratica* seedlings with *T. asperellum* ACCC30536, it was found that the xylanase secreted by the fungus not only promoted seedling growth but also induced systemic resistance, conferring broad-spectrum resistance against plant pathogens [18]. It is generally believed that plant induced resistance mainly involves signal transduction pathways mediated by salicylic acid (SA), jasmonic acid (JA) and ethylene (ET) [19]. Under *Trichoderma* treatment, the contents of JA, SA and ET in *Arabidopsis* and tomato plants increased to varying degrees, indirectly improving plant stress resistance [11]. *T. harzianum* T-22 enhances the resistance of tomato to *Cucumber mosaic* virus by upregulating the expression of SA signaling pathway genes (such as NPR1), and synergizes with the JA/ET pathways to form a broad-spectrum defense response [20]. In addition, *Trichoderma* can also promote the root development of crops by dissolving phosphorus, fixing nitrogen and synthesizing plant hormones (such as auxin), thus achieving the double effect of disease prevention and growth promotion [21]. After infecting host plants, endophytes can enhance nutrient use efficiency, especially phosphorus and potassium, contributing to improved growth [22,23].

This study focused on a biocontrol fungus, *T. harzianum* M2, to explore its protective efficacy against *Fp* infection in wheat, to analyze its ISR regulatory mechanism in wheat and its dual function of boosting growth and strengthening wheat disease resistance. This study aims to provide theoretical foundations and practical guidance for developing efficient, eco-friendly biocontrol strategies against wheat diseases, and advance sustainable agricultural production.

## 2. Results

### 2.1. Isolation and Identification of Strain

The strain M2 grew rapidly on PDA medium. Colonies were initially white with aerial mycelium at day 1 (diameter: 33.6 ± 1.2 mm). By day 4, colonies fully covered the Petri plate (diameter: 83.5 ± 1.4 mm), forming a cotton-woolly mycelial mat with a central green sporulating zone (Figure S1). Colonies were circular, white at the early stage and turned green upon sporulation; the reverse side was amber. Aerial mycelium was fast-growing, curly and white. Conidiophores were translucent and pyramidal with symmetric primary branches. Phialides were ampulliform, constricted at the base, swollen in the middle and tapering toward the apex, and arranged in two to five whorls, with conidia borne at their tips. Conidia were green, smooth-walled, and spherical to obovate (Figure S1). Based on these morphological traits, strain M2 was preliminarily assigned to the genus *Trichoderma*. For molecular phylogenetic identification, genetic loci, including *ITS*, *RPB2*, and *tef1-α*, were initially targeted for amplification and sequencing. However, repeated amplification experiments failed to obtain valid *tef1-α* gene sequences of strain M2, which was attributed to the genomic specificity of strain M2 and low matching efficiency of the universal *tef1-α* primers. Finally, a phylogenetic tree was constructed based on the *ITS* and *RPB2* gene sequences of strain M2, with *Protocrea farinosa* as the outgroup taxon (Figure S1, Table S1). The phylogenetic reconstruction clearly resolved strain M2 within the *T. harzianum* clade, showing high sequence similarity (99.56%) and robust bootstrap support with reference strains of *T. harzianum*, confirming strain M2 to be *T. harzianum*. Light-microscopy observations revealed that M2 hyphae grew along hyphae of *Fusarium pseudograminearum* (*Fp*), causing hyphal damage, loss of viability and eventual disintegration (Figure 1C–G).

### 2.2. Antagonistic Characteristics of T. harzianum M2 Against Fusarium Pathogens

Dual-culture antagonism assays revealed that strain M2 displayed strong inhibitory effects against five *Fusarium* pathogens (Figure 2A–E), including *Fp*, *Fm*, *Fc*, *Fg*, and *Fo* (Figure 2a–e). Obvious suppression of mycelial growth and altered colony pigmentation were observed on dual-culture plates. The average inhibition rates ranged from 72.35% to 91.24% (Figure 2F). Strain M2 exerted the strongest antagonistic activity against *Fp*.

### 2.3. T. harzianum M2 Showed Significant Antagonistic Activity Against Fp

To investigate whether M2 could enhance wheat resistance against *Fp*, in vitro antagonism experiments were carried out (Figure S3). It can be clearly observed that under the M2 treatment, multiple physiological indices of wheat were significantly improved. Compared with the control group, the plant height of wheat under the M2 treatment increased by 25.61% at 21 d post-inoculation; compared with the *Fp*-only group, the plant height of wheat under M2+*Fp* treatment increased by 21.46%. Furthermore, under the M2 or M2+*Fp* treatment, various indicators such as the shoot fresh weight, leaf length, root length, and root fresh weight of wheat also increased to varying degrees. These results suggest that M2 may induce systemic resistance in wheat.

### 2.4. T. harzianum M2 Significantly Promotes Wheat Growth

After 24 h of germination, wheat seeds were transplanted into hydroponic boxes and nutrient soil pots, respectively. Compared with the control group, the *Trichoderma* spore suspension at 10^5^ cfu/mL exerted the most pronounced seedling-growth-promoting effect (Figure 3A–C). Under hydroponic conditions, seedling fresh weight, leaf length, root length, and root fresh weight were all significantly enhanced under this treatment (Figure 3D–G). Relative to the control, the 10^5^ cfu/mL *Trichoderma* treatment increased seedling fresh weight by 1.92 ± 0.14 g, leaf length by 2.42 ± 0.21 cm, root length by 4.36 ± 0.33 cm, and root fresh weight by 0.36 ± 0.04 g (Table S2). On a percentage basis, seedling fresh weight, leaf length, root length, and root fresh weight were increased by 42.3%, 28.7%, 51.6%, and 35.1%, respectively. Collectively, these results demonstrate that the 10^5^ cfu/mL *Trichoderma* spore suspension strongly promotes wheat seedling growth under hydroponic conditions (Figure 3D–G).

Similar growth-promoting effects were also observed in pot experiments (Figure 3H,I). At the Trichoderma spore suspension concentration of 10^5^ cfu/mL, plant fresh weight, leaf SPAD value, root length, and leaf surface area were significantly higher relative to other treatments (Figure 3J–M, Table S3). Compared with the control, this treatment increased plant fresh weight by 0.17 ± 0.03 g (6.09%), SPAD value by 3.20 ± 0.53 (11.32%), root length by 369.84 ± 23.45 cm (16.80%), and leaf surface area by 56.41 ± 8.95 cm^2^ (30.30%). Root-scanning images revealed obvious changes in wheat root system architecture; significant increases were detected in root length, root surface area, root projection area, root volume, and average root diameter (Figure S2, Table S3). Relative to the control, root length, root surface area, projection area, volume, and average diameter were elevated by 16.80%, 45.99%, 11.47%, 5.41%, and 2.91%, respectively. Collectively, these findings indicate that the 10^5^ cfu/mL *Trichoderma* spore facilitates plant growth and development by changing root morphology, including enhanced lateral-root formation, longer and denser root hairs, and enlarged root–soil contact area, which contributes to robust plant performance.

### 2.5. T. harzianum M2 Protects Against Diseases in Glasshouse

The results of disease investigation showed that wheat seedlings infected with *Fp* showed obvious FCR disease phenotype, while wheat infected with *Fp* after M2 treatment showed almost no disease phenotypes (Figure 4A–D). Compared with the *Fp*-only group, shoot fresh weight, root length, fresh root weight and stem diameter of wheat under M2+*Fp* treatment increased by 54.48%, 21.15%, 42.28% and 30.72%, respectively (Figure 4E–H). In addition, the change trend for plant height under different treatments showed that *Fp* infection significantly inhibited the growth of wheat, while wheat under M2+*Fp* treatment was almost unaffected (Figure 4I). Here, the length of the wheat stalk lesion was used as the classification standard for FCR (Figure 4J), and the results showed that M2 treatment reduced the disease index by 58.43% and achieved 90.12% (Figure 4K, Table S4) disease control efficacy.

### 2.6. T. harzianum M2 Protects Against Diseases in the Field

In order to further explore the protection ability of M2 against FCR in wheat under field conditions, experiments were carried out in Zhoukou in 2024–2025 (Figure 5A). At the overwintering stage and early heading stage of wheat, 10^5^ cfu/mL conidial suspension was used to inoculate the roots of the wheat. It was found that there was no significant difference in the morphology of individual wheat plants among the treatments (Figure 5B–E), but the disease spot length on the stem of the wheat could reflect the different disease conditions (Figure 5F–I). The disease index was 82% in the plots treated with *Fp* and 15% in the control treatment (Figure 5J). Disease severity was calculated as the length of the lesion on the reference stem relative to seedling height. In addition, after the treatment of M2 in the field, the disease index of wheat under M2+*Fp* treatment was reduced to 28%, and the disease prevention and control rate reached 71.70% (Figure 5K,L, Table S5). A total of 100 stems were randomly selected from each plot for statistical investigation, and it was found that after M2+*Fp* treatment, most of the plants were at level 1 (Resistance) (Figure 5L). The results show that M2 pretreatment enhanced wheat immunity and improved disease resistance against FCR.

### 2.7. T. harzianum M2 Colonizes in Wheat

Strains were isolated from wheat rhizosphere soil and wheat tissues one month after treatment. *Trichoderma* was re-isolated from wheat rhizosphere soil using Bengal red culture medium, and morphological observation of the strain was performed at 5 d post-inoculation (Figure 6A,B). In addition, *Trichoderma* was re-isolated from wheat roots and stem bases on PDA medium, followed by morphological observation of the strain conducted at 6 d post-inoculation (Figure 6C,D). It was found that the colony was consistent with M2 by morphological observation of mycelium, spore and spore stalk (Figure 6E,F). Meanwhile, the process of M2 colonization in plant cells could be observed by using transgenic strains of M2. On one hand, PCR detection of GFP fragments (536 bp) in wheat root indicated that M2 had been colonized in plants (Figure 6G). On the other hand, in order to further clarify the colonization of *Trichoderma* in wheat roots, WGA-AF488 was used to observe the colonization of *Trichoderma* in wheat roots (Figure 6H–L). It could be seen that the stained wheat cell wall was red, and the cell wall of *Trichoderma* was dyed green. M2 penetrates root tissues through epidermal cells and colonizes intracellularly and intercellularly. The results showed that M2 had colonized wheat rhizosphere soil and root tissues. In conclusion, M2 hyphae intertwine within root cells, forming persistent biofilm-like structures, and exists in the vascular bundle cells of the root for a long time.

### 2.8. T. harzianum M2 Modulates Wheat Root Gene Expression During Early Colonization

The PCA results in RNA-seq showed good sample correlations, with samples clearly separated by two main eigenvectors (27.37% and 17.95%) (Figure S4). PCC analysis yielded correlation coefficients >0.98 across biological replicates (Figure S5), confirming high sequencing reproducibility. Transcriptome profiling identified 45,822, 49,099, 48,399 and 46,221 expressed genes in the control, M2, *Fp* and M2+*Fp* libraries, with 511, 1900, 815 and 867 tissue-specific genes, respectively (Figure 7A). The M2-treated group possessed the largest number of tissue-specific expressed genes.

A total of 2438 DEGs were detected between the control and M2. GO enrichment indicated enrichment in the extracellular region, cell wall and plant-type cell wall (Figure 7B). KEGG analysis highlighted plant hormone signal transduction as the predominant pathway responding to M2 elicitation (Figure 7C). WGCNA was performed on 9328 DEGs, generating seven co-expression modules (Figure 7D). The brown module was tightly associated with M2-triggered resistance (Figure 7E). GO terms including catalytic activity, single-organism metabolic process and primary metabolic process dominated the brown module (M2). The blue module (M2+*Fp*) shared partial enrichments but featured additional enrichment in cellular component organization, implying pathogen-triggered cellular remodeling (Table S6). KEGG showed that the brown module was enriched in plant hormone signal transduction, starch–sucrose metabolism and endoplasmic reticulum-associated protein processing. By contrast, the blue module was enriched in phenylpropanoid biosynthesis and plant–pathogen interaction (Table S7).

In addition, in order to more clearly understand the influence of M2 on the expression level of wheat disease resistance and defense genes, we analyzed DEGs between the M2+*Fp* group and the control. To dissect the specific protective effect of M2 under pathogen infection, we further performed a critical pairwise comparison between M2+*Fp* and *Fp* alone, which directly reflects transcriptional reprogramming triggered by M2 inoculation upon *Fp* challenge. The results showed that transition-metal ion binding (molecular function), extracellular region (cellular component), and defense response (biological process) were highly enriched GO terms with the highest enrichment of DEGs (Figure S6). Comparative GO analysis of M2+*Fp* vs. *Fp* revealed that DEGs from M2+*Fp* vs. *Fp* were significantly enriched in defense response, phenylpropanoid metabolism and hormone signaling, demonstrating that M2 potentiates host defense transcription upon *Fp* infection beyond pathogen-triggered basal immunity.

In terms of metabolic pathway enrichment analysis, apart from phenylpropanoid biosynthesis (ko00940), most differentially expressed genes were assigned to the plant hormone signal transduction pathway (ko04075). Notably, JA- and SA-dependent defense-related hormone pathways were strongly altered (Figure S7). Consistent with this, M2+*Fp* vs. *Fp* comparison further confirmed enhanced JA/SA signaling, phenylpropanoid biosynthesis and plant–pathogen interaction pathways under combined treatment. Collectively, these data suggest that M2 enhances wheat resistance by coordinating multi-hormone signaling, supporting a dual regulatory mode that maintains basal metabolism while activating defense-related pathways for systemic resistance.

### 2.9. Activation of Wheat-Induced Resistance by M2

Quantitative RT-PCR (qRT-PCR) was used to detect some general defense response genes’ expression levels, which further confirmed the significant upregulation of SA, JA and general defense response genes under M2 treatment (Figure 8). Here, we selected 12 disease-responsive genes from RNA-seq to detect *Trichoderma*-induced resistance in wheat. These defense response genes were independently selected based on the published literature. The identified disease-responsive genes were functionally categorized into four regulatory modules: (1) jasmonate signaling components encompassing the JA biosynthetic enzymes lipoxygenase *TaAOS1* and phenylalanine ammonia-lyase *TaPAL*, with lipase *LIPASE* and transcription factor *TaWRKY78* participating in JA signal amplification [24]; (2) salicylate-mediated systemic acquired resistance (SAR) machinery involving pathogenesis-related protein *PR2* and regulatory cofactor *TaNPR1* [25]; (3) abiotic stress integrators, including ABA biosynthetic enzymes 9-cis-epoxycarotenoid dioxygenase *NCED* and violaxanthin de-epoxidase *VDE1*, alongside ethylene biosynthesis regulator *TaPIE1* [26]; and (4) core defense and senescence markers, with chitinase *TaCHI1* and ubiquitin ligase *WCI3* constituting basal immunity, and senescence-associated gene *SAG3* serving as programmed cell death indicator [27]. Transcriptomic profiling revealed that M2 treatment significantly activated defense-related gene expression across multiple pathways, except for the pathogenesis-related gene *PR2* and ethylene biosynthesis gene *TaPIE1*. Strikingly, under M2+*Fp* treatment, all twelve defense marker genes exhibited significant differential expression (|log2FC| > 1, FDR < 0.05), including strong induction of *PR2* and *TaPIE1*. These expression dynamics suggest a biphasic regulatory mechanism of M2-induced systemic resistance, characterized by the priming phase under mono-treatment and synergistic potentiation during pathogen challenge.

### 2.10. T. harzianum M2 Colonization Can Improve the Uptake Efficiency of Soil Nutrients by Plants

The results showed that M2 could effectively promote the uptake of soil phosphorus content and dry matter accumulation in wheat, but there was no significant difference in the uptake of nitrogen and potassium between the control group and M2 treatment group (Figure 9A–D). The yield comparison experiment showed that the theoretical yield of wheat after M2 treatment increased by 8.15% compared with the Control group. At the same time, compared with the *Fp*-only group, wheat yield under M2+*Fp* treatment increased by 4.92%, and the increase in yield was mainly achieved by increasing thousand-grain weight (Figure 9E). In addition, the preliminary test results showed that M2 treatment increased the shoot fresh weight of wheat seedlings. It is speculated that the colonization of M2 in the root can enhance the uptake efficiency of nutrients in wheat, thereby achieving the goal of increasing yield.

## 3. Discussion

### 3.1. T. harzianum M2 Is a Novel Trichoderma Strain with Great Potential for Biocontrol

FCR, mainly caused by *Fusarium* spp., is an important disease that seriously threatens wheat yield and quality [28]. Among them, *Fp* is the dominant population of FCR in the North China Plain [29]. In this study, strain M2 exhibited typical *Trichoderma* morphological traits on PDA, including fast-growing white aerial mycelia, green conidial zones and distinctive sporulating structures. These phenotypes match well with the classic taxonomic description of *T. harzianum* [30], which is further supported by the diagnostic amber pigment on the colony reverse [31]. M2 fully covered the Petri dishes within 3 d and rapidly produced mature conidia, indicating strong competitive capacity and promising biocontrol potential for future agricultural disease-control research [15]. The pathogens used in this study were isolated in field and have high infectivity for FCR in seedling stage. Therefore, *Fp* was used to explore the mechanism of *Trichoderma*-induced wheat resistance to FCR. Currently, the available biocontrol agents against *Fusarium* spp. are generally considered to be primarily based on antagonism [32], as earlier studies prioritized antagonistic effects while neglecting induced-resistance pathways. Nevertheless, biocontrol agents are increasingly acknowledged as plant-defense priming agents that activate systemic resistance and improve crop yield [33]. Our hydroponic and pot-culture assays demonstrated that M2 is well adapted to diverse rhizosphere microenvironments and stably promotes root biomass accumulation across different culture systems, highlighting its growth-promoting capability. We speculate that M2 may trigger systemic plant responses via secreted soluble factors or volatile organic compounds, as exemplified by 6-amyl-2H-pyran-2-one (6-PP) from *T. asperellum* T34, which modulates phytohormone signaling to reshape root architecture [34]. It should be noted, however, that our current study has not performed metabolite profiling or functional validation of individual M2-derived metabolites. Thus, the involvement of soluble secretory compounds and VOCs remains a plausible but unproven mechanism, and further work is required to identify the priming-active metabolites from strain M2. Recent studies have emphasized the necessity of functional and ecological validation for specialized metabolites in *Trichoderma* spp., providing important context for distinguishing the biological effects of living *Trichoderma* cells from actions specifically assigned to fungal metabolites [35,36,37]. Strikingly, M2 harbors strain-specific features distinct from many commercial *T. harzianum* strains that depend heavily on mycoparasitism: its beneficial effects are largely attributed to host immune reprogramming instead of simple pathogen suppression [38]. Unlike some *Trichoderma* isolates that trigger persistent constitutive defense even in the absence of pathogens, M2 avoids excessive immune activation under pathogen-free conditions and alleviates fitness costs from growth-defense trade-offs [39]. Collectively, M2 represents a promising novel *T. harzianum* isolate for biocontrol applications.

### 3.2. T. harzianum M2 Can Enhance the Resistance of Wheat to FCR

*Trichoderma* spp. are ubiquitous biocontrol fungi in nature, among which *T. harzianum* has received considerable attention for its capacity to suppress diverse crop-pathogenic microorganisms [40]. For instance, *T. harzianum* EMF910 inhibits pathogens responsible for *Astragalus* root blight and reduces disease incidence in saline soils of Ningxia [41], while *T. harzianum* T9131 enhances astragalus resistance against the Hunyuan-derived pathogen HYFS-1 [42]. Beyond its efficacy against soil-borne diseases, *T. harzianum* also controls postharvest and other fungal plant diseases; *T. harzianum* Tha739, for example, exerts satisfactory biocontrol against apple bitter rot [43]. In the present study, *T. harzianum* M2 exhibited prominent biocontrol efficacy against FCR, with dual functions in plant growth promotion and disease resistance.

These findings align with the broad-spectrum biocontrol properties reported for *Trichoderma*. *Trichoderma* can directly constrain pathogen proliferation via secreted chitinases and polyketones, and simultaneously trigger plant systemic resistance [30]. The protective effects of M2 may also rely on modulation of plant immune signaling cascades [44]. Previous work has demonstrated that *Trichoderma* boosts host resistance by modulating ROS homeostasis, promoting callose deposition, and upregulating defense-related genes. For example, *T. virens* elevates wheat peroxidase and superoxide dismutase activities to facilitate ROS scavenging and restrict pathogen spread [45]. The reduced stem lesion length observed in M2-treated wheat in our study may involve analogous immune-related mechanisms [46]. We further assessed the activation of induced systemic resistance by profiling several canonical defense-response genes, including *TaAOS1, TaPAL* and *TaWRKY78*. Compared with a previous study on *Purpureocillium lilacinum* YZ1 [47], our investigation on the novel isolate M2 incorporated additional marker genes for validation, achieving improved reliability and broader coverage in gene-expression assays. Collectively, these results support M2 as a promising agent to improve wheat resistance to FCR.

Nevertheless, a notable efficacy gap exists between greenhouse and field environments. M2 achieved substantial disease suppression under controlled greenhouse conditions, yet its performance moderately declined under field conditions [48]. Such variability is common for *Trichoderma*-based biocontrol agents, largely attributable to fluctuating soil conditions, inter-microbial competition, and uneven rhizosphere colonization. Complex field rhizosphere environments restrict *Trichoderma* population establishment, in contrast to favorable greenhouse substrates that sustain robust colonization. Accordingly, biocontrol candidate screening should prioritize rhizosphere adaptability rather than merely high potency under greenhouse settings [49].

### 3.3. Colonization of M2 Induces Systemic Resistance in Wheat

Rhizosphere microorganisms, including *Trichoderma* spp., are critical for plant growth and fitness, and efficient rhizosphere colonization serves as a prerequisite for their beneficial functions [50]. The growth-promoting performance of such fungi is frequently constrained by their rhizosphere colonization competence [51]. In this study, M2 exhibited sustained rhizosphere persistence in wheat, formed stable biofilms, and penetrated root epidermal cells to reach xylem vessels, matching well with well-documented *Trichoderma* colonization strategies [52]. Combining GFP labeling and WGA-AF488 staining, we visualized the interwoven distribution of M2 mycelia within wheat root tissues, offering direct cytological evidence for the *Trichoderma*–host interaction interface [53]. The split-root system isolated the local colonization area of M2 and the uninoculated systemic area of wheat roots. The differential expression of disease resistance and phosphorus uptake genes in the uninoculated root area fully proves that M2 can induce systemic resistance and root developmental regulation in wheat, rather than only local colonization effects. Transcriptomic profiling revealed profound transcript reprogramming in wheat roots upon M2 exposure. Differentially expressed genes were enriched in plant-hormone signal transduction, carbohydrate metabolism, and endoplasmic reticulum protein processing. Consistently with prior studies, hormone-signaling reprogramming is tightly linked to *Trichoderma*-triggered SA- and JA-dependent defense responses [54,55]. Enrichment of gene terms associated with extracellular compartments and cell wall functions further implies the involvement of *Trichoderma*-secreted cell wall-degrading enzymes such as chitinases and glucanases [56]. WGCNA identified the brown module as strongly associated with host resistance; enrichment for catalytic activity and primary metabolism supports a model whereby M2 boosts immunity by remodeling host metabolic networks, consistent with reported *Trichoderma*-mediated basal defense activation [34]. In this study, the biphasic regulation mode of M2 on wheat defense genes was verified by qRT-PCR, and the molecular basis of ISR was revealed. Our findings indicate that M2 establishes a classical defense-priming state in wheat. In the absence of pathogens, M2 pre-conditions JA-dependent signaling without triggering costly full-scale defense outputs. When challenged by *Fp*, primed wheat rapidly amplifies SA-JA-ET immune cascades to mount robust resistance. This priming mechanism minimizes metabolic trade-offs under pathogen-free conditions while permitting rapid defense deployment upon infection, providing a plausible explanation for dual capacity to promote growth and improve disease resistance [57,58]. Such a response aligns with canonical *Trichoderma*-elicited ISR, which potentiates host responsiveness to subsequent pathogen attack [59]. Supporting this model, M2-driven upregulation of *TaAOS1* and *TaPAL* activates JA biosynthesis [24], while induced *TaWRKY78* reinforces JA-signal amplification via *WRKY* transcription factors [60]. In comparison with other well-characterized *Trichoderma* strains reported previously, strain M2 displays distinctive advantages in remodeling wheat root morphological traits and activating long-distance systemic resistance spanning root and shoot tissues [61,62]. Practically, this ISR-associated trait confers dual beneficial functions, including enhanced resistance to Fusarium crown rot and improved tolerance to multiple abiotic stresses [63]. Such characteristics highlight promising prospects for deploying M2-mediated ISR in sustainable biocontrol strategies targeting wheat soil-borne diseases under complex field environments [64,65]. Taken together, M2 pretreatment elicits wheat ISR via coordinated activation of multiple hormone-dependent defense pathways.

### 3.4. T. harzianum M2 Remodels Root Architecture and Enhances Phosphorus Accumulation in Wheat

*Trichoderma* represents a key group of biocontrol and growth-promoting agents for modern agriculture. Its yield-boosting potential largely stems from improved plant nutrient acquisition, which is tightly linked to rhizosphere ecological modulation [66]. As saprophytic fungi, *Trichoderma* spp. degrade recalcitrant soil organic substrates such as cellulose and hemicellulose to sustain rhizosphere survival [67]. For example, *T. harzianum* drives straw–carbon conversion into soil organic carbon via extracellular enzyme secretion and stable root colonization, thereby sustaining soil nutrient homeostasis [68]. Nevertheless, beneficial outcomes of *Trichoderma* are frequently constrained by its rhizosphere colonization competence [51]. Certain *Trichoderma* isolates mobilize insoluble soil phosphate through organic-acid excretion (such as oxalic acid and citric acid) [69]. It is worth noting that M2+*Fp* treatment can inhibit pathogens while still maintaining phosphorus uptake advantages, suggesting that M2 has the dual function of synergistic disease resistance and growth promotion [70]. RNA-seq uncovered core gene regulatory events during host colonization, pointing to early-stage activation of plant defense responses. A comparable pattern has been reported for *Bacillus amyloliquefaciens* SQR9, whose colonization triggers immune-related transcription factors (*WRKY29*, *ERF1B*) together with auxin signaling in cucumber roots [71]; this illustrates a universal requirement for beneficial microbes to balance host defense and growth programs. Consistent with this, M2 is capable of enhancing wheat phosphorus accumulation by inducing phosphate-transporter gene expression in roots [72]. Sustained M2 colonization further supports durable pathogen suppression and continuous nutrient uptake, lowering reliance on agrochemical inputs [73]. In the case of M2, its ability to modulate such colonization-related genes not only enhances *Trichoderma*’s rhizosphere persistence but also optimizes the trade-off between host immune activation and nutrient-acquisition-related physiological processes. Collectively, the present study demonstrates that *T. harzianum* M2 achieves FCR suppression via colonization and sophisticated defense priming rather than simple antagonistic action [39]. The structural changes in the wheat root system induced by M2 may contribute to the observed increase in phosphorus accumulation by increasing the root–soil interface. Its strain-specific biphasic immune regulation reconciles disease resistance and plant growth, yet performance attenuation from greenhouse to field reminds us that rhizosphere competitiveness remains a major practical bottleneck [49]. Further work is required to characterize M2-derived priming metabolites, optimize field application formulations, and dissect its interaction with the native soil microbiome, to accelerate the practical translation of M2 into sustainable wheat production.

## 4. Materials and Methods

### 4.1. Strains and Plants

M2 (GenBank accession No. PV802261) was isolated from the soil using Bengal red culture medium (Cat no. R8200, Solarbio, Beijing, China) in Zhoukou National Agricultural High-tech Industry Demonstration Zone, Henan Province, China. This strain was phenotypically identified as a species of the genus *Trichoderma*. Local strains of *Fp* (GenBank accession No. PV810307) were used in this study, and molecular identification of *Fp* has been completed using specific primers [74]. Primer sequences are listed in Table S8. The semi-winter wheat Zhengmai 1860 was used in this study, and the seeds were bred and preserved in our lab. The soil from Pindstrup (www.pindstrup.com/ (accessed on 21 May 2026)) was used for all the glasshouse experiments. Prior to pot experiments, the commercial Pindstrup substrate was autoclaved twice at 121 °C for 20 min with a 24 h interval between cycles to eliminate indigenous fungal propagules. Disease-resistance assays were performed at our laboratory’s core experimental field (33°38′ N, 115°13′ E).

For molecular identification of Trichoderma strains, the Neighbor-Joining (NJ) method in MEGA 7.0 software (http://www.megasoftware.net/ (accessed on 21 May 2026)) was applied to generate the phylogenetic tree. Bootstrap analysis with 1000 replicates was carried out to assess topological reliability, and the phylogenetic tree was visualized in MEGA 7.0.

### 4.2. Growth Media and Microbial Cultivation Conditions

For culture of *Trichoderma* spp. and *Fp*, potato dextrose agar (PDA) was prepared by dissolving 46 g PDA powder (Cat no. P8931, Solarbio, Beijing, China) in 1 L of sterile water. For spore production of *T. harzianum* M2, the strain was grown in potato dextrose broth (Cat no. P9240, Solarbio, Beijing, China), and the pH was adjusted to 5.0. Mass production of *Fp* spores was performed using 6% mung bean soup medium [9]. Millet medium was used for solid propagation of pathogenic spores [75]. All media were autoclaved at 121 °C for 20 min using an autoclave (Model LDZM-80L-I, Shenan Instrument Co., Shanghai, China).

### 4.3. Antagonistic Activity of T. harzianum M2 Against the Pathogenic Fusarium Species

As PDA supports the growth of diverse plant-pathogenic *Fusarium* species, the in vitro antagonistic activity of M2 against *Fp*, *Fg*, *Fo*, *Fc*, and *Fm* was determined using dual culture assays on PDA. Mycelial plugs 5 mm in diameter were obtained using a sterile cork borer. The mycelial plug (5 mm diameter) was placed 1/4 of the plate radius from the plate edge, and the antagonism was observed by measuring the inhibition zone. The inhibition rate was calculated as: (colony radius of control group − colony radius of treatment group)/colony radius of control group × 100%. All experiments were repeated three times, with three replicate plates per confrontation assay.

### 4.4. Hydroponic Culture Assay

Wheat seedlings were cultured in a hydroponic system using modified Hoagland’s nutrient solution. Seeds were surface-sterilized and pre-germinated on moist filter paper under dark conditions. Uniform germinated seedlings were transferred to hydroponic containers filled with continuously aerated nutrient solution. The nutrient solution was renewed every 3 days to maintain nutrient availability and pH stability.

M2 spore suspensions were prepared by harvesting spores from 7-day-old PDA cultures, followed by filtration through two layers of sterile gauze to remove mycelial fragments. Spore concentration was quantified using a hemocytometer, and spore suspensions were diluted to 10^3^, 10^5^, and 10^7^ cfu/mL. Different concentrations of M2 spore suspensions were applied to the hydroponic solution to inoculate wheat root systems. Seedlings cultured in nutrient solution supplemented with an equal volume of sterile water served as the uninoculated control. The hydroponic experiment was performed with three biological replicates for each treatment. Each biological replicate of the hydroponic experiment contained 16 uniform wheat seedlings.

### 4.5. Beneficial Effects of T. harzianum M2 Tested in Pot-Culture Experiments

To investigate the beneficial effects of M2 on wheat, glasshouse experiments were conducted with autoclaved soil. When wheat seedlings reached the three-leaf stage in pots (16 cm × 16 cm × 16 cm pots, 600 g of soil/pot), the experimental treatments began. M2 was applied as a spore suspension at a concentration of 10^5^ cfu/mL. Spores were directly applied to the roots of the plants, and there were nine wheat seedlings in each treatment, with 10 mL of spore solution for each seedling. The cultivation of *Fp* was carried out according to previous studies [74]. Millet medium was used for solid propagation of *Fp* spores. The detailed methods can be found in previous studies [76]. The experiment involved four treatments, including the control (mock inoculated), M2 only, *Fp* only, and M2+*Fp*. Each treatment had nine wheat seedlings. Ten days after being treated with M2, *Fp* inoculum was applied to the M2+*Fp* group. All treatments were repeated three times independently.

### 4.6. Disease Rating of Fp Infection Under Glasshouse Conditions

*Fp* was inoculated in the millet medium and continuously cultured for 15 days. Furrows were dug between the sowing rows (1 m long and 10 cm deep). After thoroughly mixing the infected millet grains with dry soil, the mixture was evenly scattered in the ditches. They were then gently covered with soil and moistened with sterile water. The incidence of FCR in each treatment was investigated every 7 days after treatment. No fewer than 200 plants per treatment were randomly selected for disease resistance identification, including all tillers of each plant. The disease severity was evaluated according to the degree of wheat leaf sheath infection. Disease-grading criteria were defined as follows: Grade 0, no visible necrotic or brown symptoms on leaf sheaths; Grade 1, scattered small brown spots covering <10% of the leaf sheath area; Grade 2, brown lesions covering 10–30% of the leaf sheath area; Grade 3, obvious browning covering 30–50% of the leaf sheath area; Grade 4, extensive browning covering 50–80% of the leaf sheath area; Grade 5, severe browning, sheath necrosis or plant death. Disease index (DI) = (0 × X0 + 1 × X1 + 2 × X2 + 3 × X3 + 4 × X4 + 5 × X5)/[(X0 + X1 + X2 + X3 + X4 + X5) × 5] × 100%. In the formula, 0, 1, 2, 3, 4, and 5 represent different disease grades, while X0, X1, X2, X3, X4, and X5 indicate the number of wheat plants corresponding to each disease grade. Relative disease index (RDI) = 100 × 1 − (DI _max value_ − DI)/DI _max value_)). Disease control effect = Average (∑RDI). The detailed methods can be found in previous studies [77,78].

### 4.7. Field Experiment Arrangement and Disease Identification

Field experiments were conducted in the core experimental field of Zhoukou National Agricultural High-tech Zone, Henan Province (33°38′ N, 115°13′ E) (2024–2025), with a randomized block design. Each treatment contained three independent replicate plots (10 m × 8 m per plot). *Fp* inoculum (10^5^ cfu/mL wheat straw inoculant) was uniformly spread on topsoil before sowing. For T. *harzianum* M2 treatment, 10^5^ cfu/mL M2 conidial suspension was irrigated to wheat roots at the overwintering and early heading stages. All plots received consistent field management. No additional chemical fungicides were applied in the whole trial period to eliminate the interference of pesticide factors on the experimental results. The field water supply relied on natural rainfall combined with regular supplementary irrigation to ensure consistent soil moisture and irrigation conditions among all plots. One hundred wheat stems were randomly sampled per plot for disease assessment. For identification of DI, RDI and disease effect, consistent formulas were applied to greenhouse and field data [77,78].

### 4.8. Split-Root Experiment

For the induction resistance assay, a split root assay was chosen to verify the interaction with wheat roots between M2 and *Fp* on wheat roots. Each split-root pot contained two isolated root compartments filled with twice-autoclaved Pindstrup substrate; wheat root systems were manually separated into two equal portions and placed into individual compartments without physical cross-compartment substrate mixing. This split-root cultivation system can effectively isolate the growth space of root tissues on both sides, which is a reliable and appropriate experimental method to explore the systemic defense response of wheat plants and verify the systemic interaction between M2 and *Fp* without physical contact. Wheat seedlings were pre-inoculated with M2 spore suspension (10^5^ cfu/mL) on one side of the split root, and with *Fp* inoculum on the opposite compartment 3 d later. Wheat seedlings were pre-inoculated with M2 spore suspension (10^5^ cfu/mL) on two sides of the split root, and with *Fp* inoculum on one side 3 d later. In this experiment, we set up a strict unilateral fixed treatment layout for the split-root system, which ensured complete spatial separation of the two treatments and avoided mutual interference. All experimental treatments were independently repeated with three biological replicates, and each biological replicate contained a complete set of all treatment conditions involved in the experiment, including the M2 unilateral treatment + *Fp* unilateral treatment group and the full *Fp* treatment control group. Meanwhile, the placement of different treatment pots and the left–right orientation of split-root compartments were completely randomly allocated in the greenhouse to eliminate positional deviation and experimental bias. All pots were randomly arranged in a greenhouse with consistent temperature (22 °C), a 16 h light/8 h dark photoperiod and 50% relative humidity. Root samples were collected at 48 h post-pathogen inoculation; the samples were stored at −80 °C for RNA-seq.

### 4.9. Observation of T. harzianum M2 Colonization in Wheat Roots

Using Bengal red culture medium and PDA medium, M2 was re-isolated and identified from wheat rhizosphere soil and wheat tissues one month after *Trichoderma* treatment. The root and leaf tissues of the wheat were collected for PCR detection using *ITS*, *RPB2* and genes. In addition, WGA-AF488 (catalog No. W11261, Thermo Fisher Scientific, Waltham, MA, USA) and propidium iodide (catalog No. P4170, Sigma-Aldrich, St. Louis, MO, USA) (1 µg/mL PI, 10 µg/mL WGA-AF488; 0.02% Tween-20 in PBS, pH 7.4) were used to stain fungal cell walls and plant cell walls. Root samples were fixed in 4% paraformaldehyde, washed three times with PBS (pH 7.4), and incubated in the mixed staining solution for 40 min at room temperature in darkness, followed by three PBS rinses before microscopic observation.

Green fluorescent protein (GFP) fragment was inserted between two restriction sites (SalI/KpnI) of the original vector pCEV-G4-Km, which carries the kanamycin resistance gene as the selection marker. Then, the obtained pCEV-GFP vector was transferred into the wild-type M2 strain by PEG-mediated protoplast transformation [79]. Transformants were first screened on selective medium supplemented with kanamycin. Putative positive transformants were further verified by PCR amplification of the GFP gene, and GFP fluorescence signal was detected using a Nikon confocal microscope (AX NIS-Elements 5.4, Tokyo, Japan) to confirm GFP expression. To evaluate genetic stability of the GFP-labeled M2 strain, the positive transformant was sub-cultured for at least 5 successive generations without antibiotic selection, and the maintenance of the GFP gene and stable fluorescent signal were re-confirmed by PCR and confocal microscopy observation. All fluorescence images were acquired using the above-mentioned confocal system. Stained samples were observed simultaneously under a confocal microscope using excitation light at 488 nm and 561 nm. Detailed data can be found in Kimotho’s descriptions [47]. The primers of GFP gene fragments are shown in Table S6.

### 4.10. RNA-Seq and Bioinformatics Analyses

An RNA-Seq experiment was conducted to explore the effects of *T. harzianum* M2 or pathogen treatment on the growth of wheat roots, and each treatment consisted of three individual hydroponic boxes, with each containing nine seedlings. The experiment was conducted using the improved Hoagland nutrient solution (1.5 mM Ca(NO_3_)_2_, 5 mM KNO_3_, 2 mM NaNO_3_, 1 mM MgSO_4_, 1 mMKH_2_PO_4_, 25 μM FeEDTA, 160 nM CuCl_2_, 9.2 μM H_3_BO_3_, 3.6 μM MnCl_2_, 16 nM Na_2_MoO_4_, 5 μMKCl, and 770 nM ZnCl_2_, with pH adjusted to 5.8). When the seedling coleoptile grew to 1 cm, the seedlings were transferred to the hydroponic box. Then the seedlings were subjected to experimental treatment carefully (Control, M2, *Fp*, and a combination of M2 and *Fp*). The plants were maintained in a growth chamber at 22 °C and 50% relative humidity, and the light cycle was 16 h of light and 8 h of dark. Roots were washed thoroughly four times to eliminate extraradical hyphae and harvested at 48 h post inoculation. Each treatment included three independent biological replicates.

A total of 12 root samples were collected promptly in liquid nitrogen for RNA extraction, which was later used for RNA-Seq analysis and qRT-PCR. RNA-Seq library construction and sequencing were performed by Sangon Biotech (Shanghai) Co., Ltd. (Shanghai, China) (https://www.sangon.com/ (accessed on 21 May 2026)). The wheat genome version IWGSC_RefSeq_v1.0 (https://www.ensembl.org/genome-selector/search?species_taxonomy_id=4565/(accessed on 21 May 2026))was used for sequence alignment. The false discovery rate (FDR < 0.01) and fold change (FC ≥ 2) were set as the thresholds for DEGs. The Pearson correlation coefficient (PCC) and principal component analysis (PCA) were used to assess the correlation among biological replicates [80,81]. Detailed methods for RNA-Seq and relevant bioinformatics analyses can be found in previous studies [82]. The BioProject accession of raw transcriptomic data in the National Center for Biotechnology Information (https://www.ncbi.nlm.nih.gov/ (accessed on 21 May 2026)) is PRJNA1247731.

### 4.11. qRT-PCR for Quantifying Wheat Genes

The root and leaf samples of the control, M2 only, *Fp* only, and M2+*Fp* were prepared for qRT-PCR. Samples used for qRT-PCR were identical to those used for RNA-Seq. All primers were designed using Primer Premier 5.0 software (https://www.premierbiosoft.com/ (accessed on 21 May 2026)). Information on the primers is listed in Table S6. The detailed methods of reverse transcription and qRT-PCR can be found in previous studies [83]. The reaction of quantitative qRT-PCR was performed in 20 μL volumes. The wheat *actin* gene was used as an internal control with three biological replicates. The gene expression levels were calculated according to the 2^−∆∆Ct^ method [84].

### 4.12. Plant Nutrient Content Determination

Grain yield-related traits, including spikes per hectare, grains per spike and 1000-grain weight, were measured at harvest [85]. Wheat plants in the field at the harvest stage were selected, deactivated at 105 °C for 30 min, dried to constant weight at 80 °C, and crushed and digested after weighing, and the content of total nitrogen in wheat plants was determined by Kjeldahl nitrogen analyzer [86]. The determination of phosphorus and potassium content in wheat was carried out mainly via previous research methods [87].

### 4.13. Statistical Analysis

All statistical analyses were re-examined to match actual dataset processing. For two-group comparisons, the unpaired Student’s *t*-test was applied. For multiple-group comparisons (≥3 groups), one-way analysis of variance (ANOVA) followed by Tukey’s honestly significant difference post hoc test was performed. Letter annotations in all figures were generated strictly based on ANOVA–Tukey outputs. Different lowercase letters indicate statistically significant differences at *p* < 0.05. *p* < 0.05 was considered to be statistically significant. All quantitative data are presented as mean ± SD (standard deviation). Statistical analyses and mapping were completed with GraphPad Prism v10 (GraphPad Software, Boston, MA, USA).

## 5. Conclusions

This study characterizes *T. harzianum* M2 as a potential biocontrol agent against wheat crown rot caused by *F. pseudograminearum*. Strain M2 displays strong antifungal activity, remodels root architecture and colonizes wheat root tissues. It may induce systemic resistance via multi-hormone pathways and exhibits disease control effects in greenhouse and field tests. Moreover, M2 facilitates wheat phosphorus uptake to improve plant biomass and yield. These findings provide insights for developing strategies for green wheat production and sustainable agriculture, though further work is needed to better clarify its systemic resistance, root colonization performance and agronomic application potential.

## Figures and Tables

**Figure 1:**
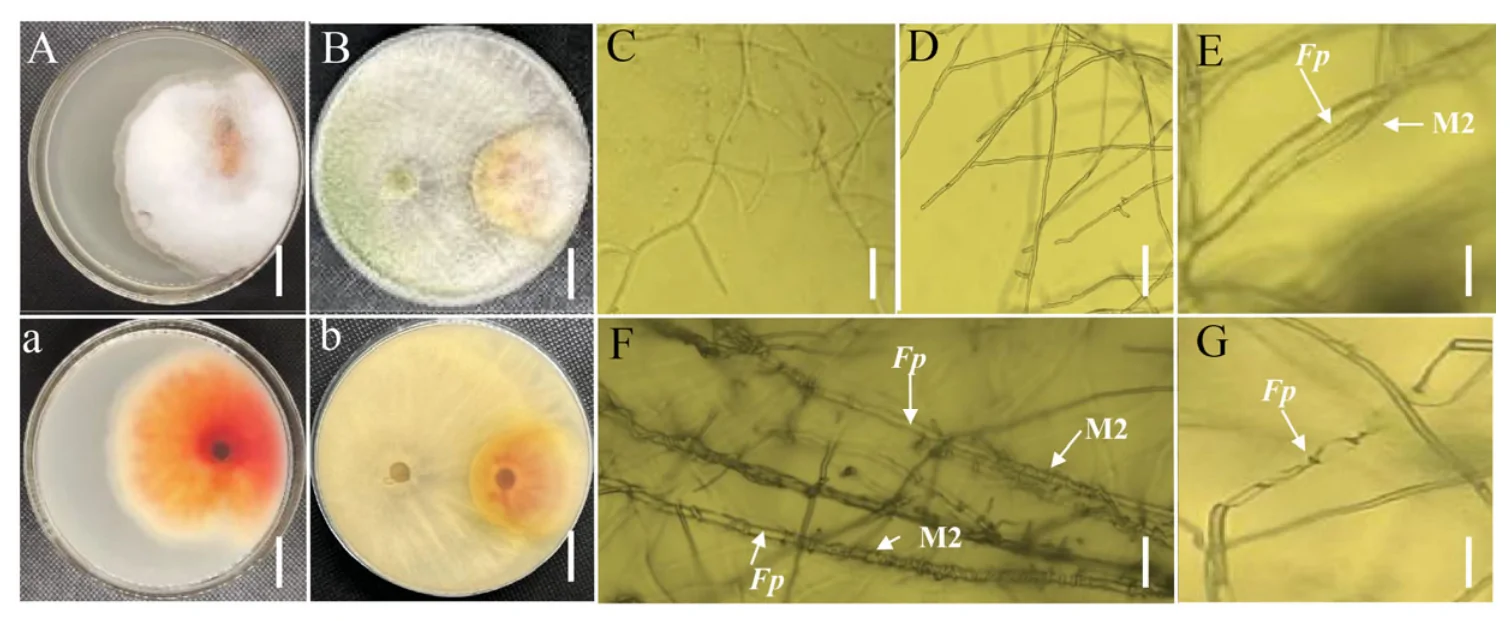
Effects of M2 on mycelial growth of *Fp.* (**A**,**a**) The morphologic map of *Fp*; (**B**,**b**) the confrontation cultivation of M2 and *Fp*; (**C**) the mycelium of M2 under normal growth; (**D**) the mycelium of *Fp* under normal growth; (**E**) M2 and *Fp* are close; (**F**) M2 mycelium growth wrapped around *Fp*; (**G**) the hyphal breakage of *Fp*. Scale bars: 2 cm (**A**,**B**,**a**,**b**); 20 μm (**C**–**G**).

**Figure 2:**
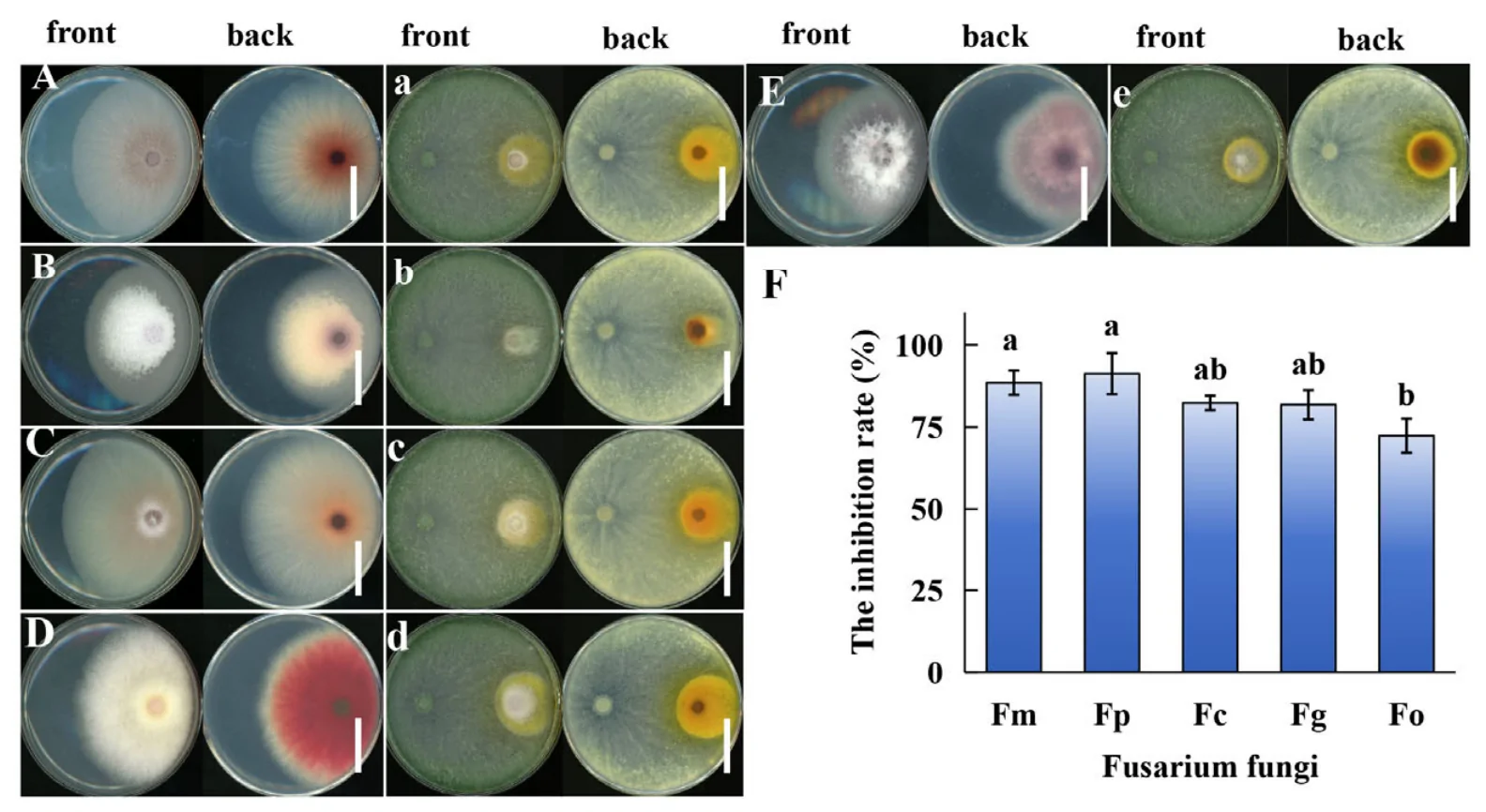
Morphological observation (front and back) of M2 against *Fusarium* fungi at day 5. (**A**–**E**) Morphology of different pathogens in the control group; (**a**–**e**) observation of M2 against *Fusarium* fungi. (**F**) The antagonism rate of M2 against *Fusarium* fungi. Scale bars: 4 cm (**A**–**E**,**a**–**e**). Error bars represent the SE of means. All experiments were repeated three times, with three replicates. Different letters at the top of the bars indicate significance (*p* < 0.05, one-way ANOVA followed by Tukey’s multiple comparison test, n = 3).

**Figure 3:**
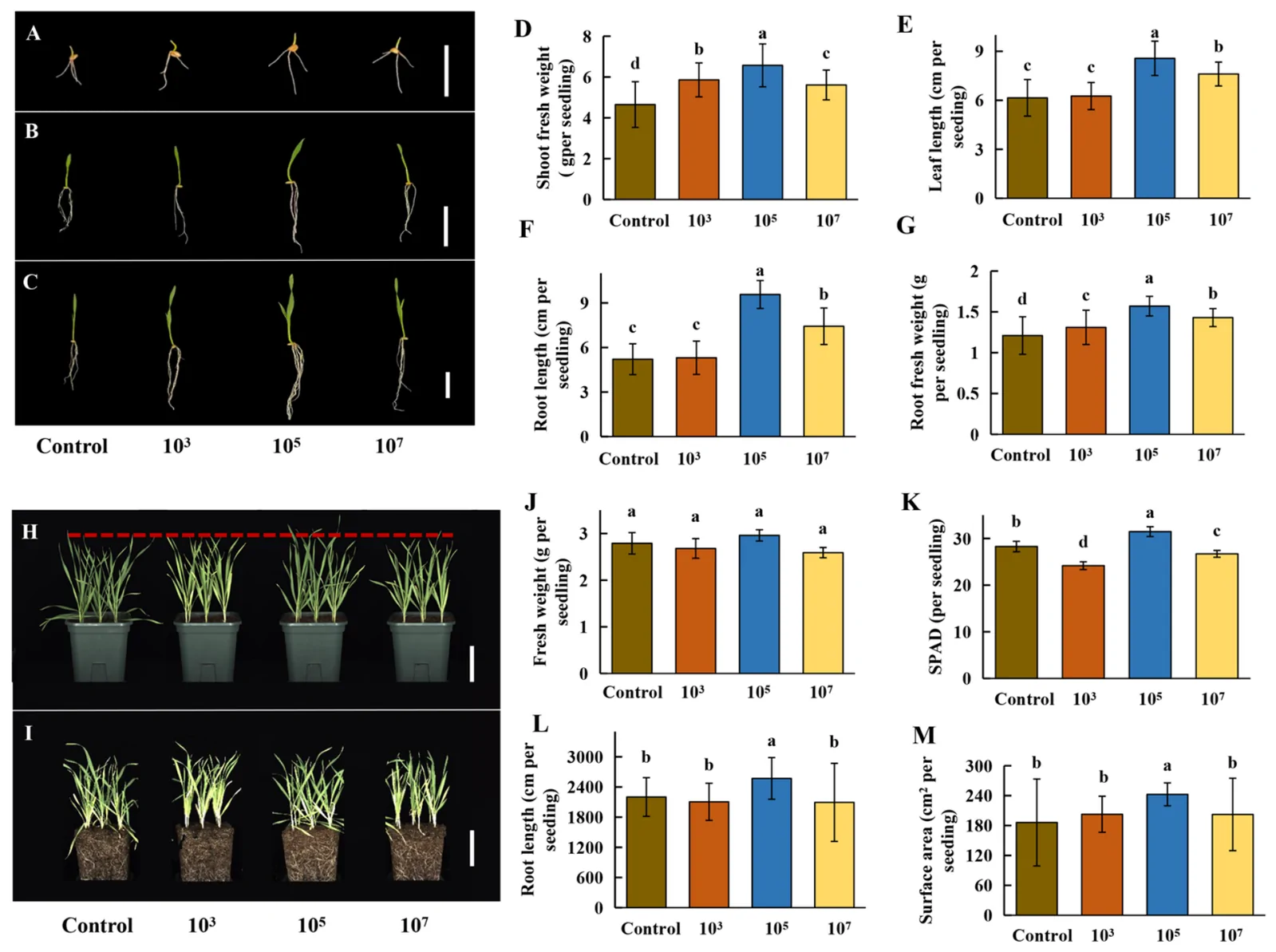
M2 inoculation promotes wheat growth in the hydroponic condition and pot condition (n = 10, respectively). (**A**–**C**) Comparison of M2-inoculated plants with non-inoculated plants on the first, second and third days, respectively; (**D**) shoot fresh weight at day 3; (**E**) leaf length at the third days; (**F**) root length at day 3; (**G**) root fresh weight at day 3; (**H**) comparison of M2-inoculated plants with non-inoculated plants at day 15; (**I**) wheat root morphology in the soil profile; (**J**) fresh weight per seedling at day 15; (**K**) SPAD values at day 15; (**L**) root length at day 15; (**M**) surface area of root at day 15. Scale bars: 1 cm in (**A**–**C**); 10 cm in (**H**,**I**). All experiments were repeated three times. Different letters indicate significant difference (*p* < 0.05, one-way ANOVA followed by Tukey’s multiple comparison test, n = 3).

**Figure 4:**
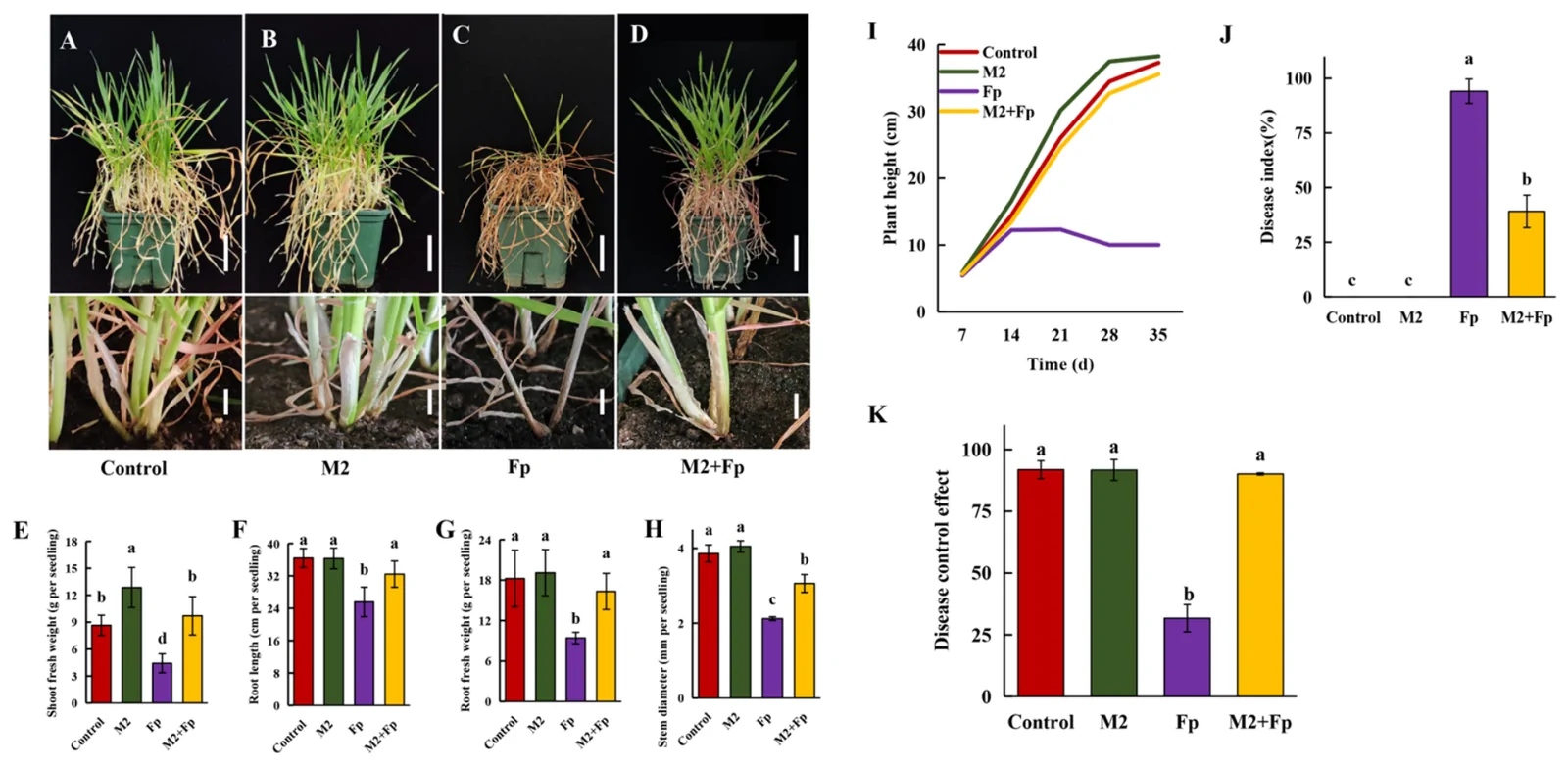
Resistance test of the M2 strain under different treatments in the greenhouse. (**A**–**D**) Phenotype of wheat seedlings (up) and roots (down); (**E**) shoot fresh weight of wheat; (**F**) root length of wheat; (**G**) root fresh weight of wheat; (**H**) stem diameter of wheat; (**I**) change trend for plant height (n = 3, respectively); (**J**) stem lesion length of wheat, browning or wilting caused by natural aging (n = 20, respectively); (**K**) disease control effect of wheat (n = 200, respectively). Scale bars: 5 cm in (**A**–**D**). Error bars represent the SE of means. All experiments were repeated three times. Different letters at the top of the bars indicate significance (*p* < 0.05, one-way ANOVA followed by Tukey’s multiple comparison test).

**Figure 5:**
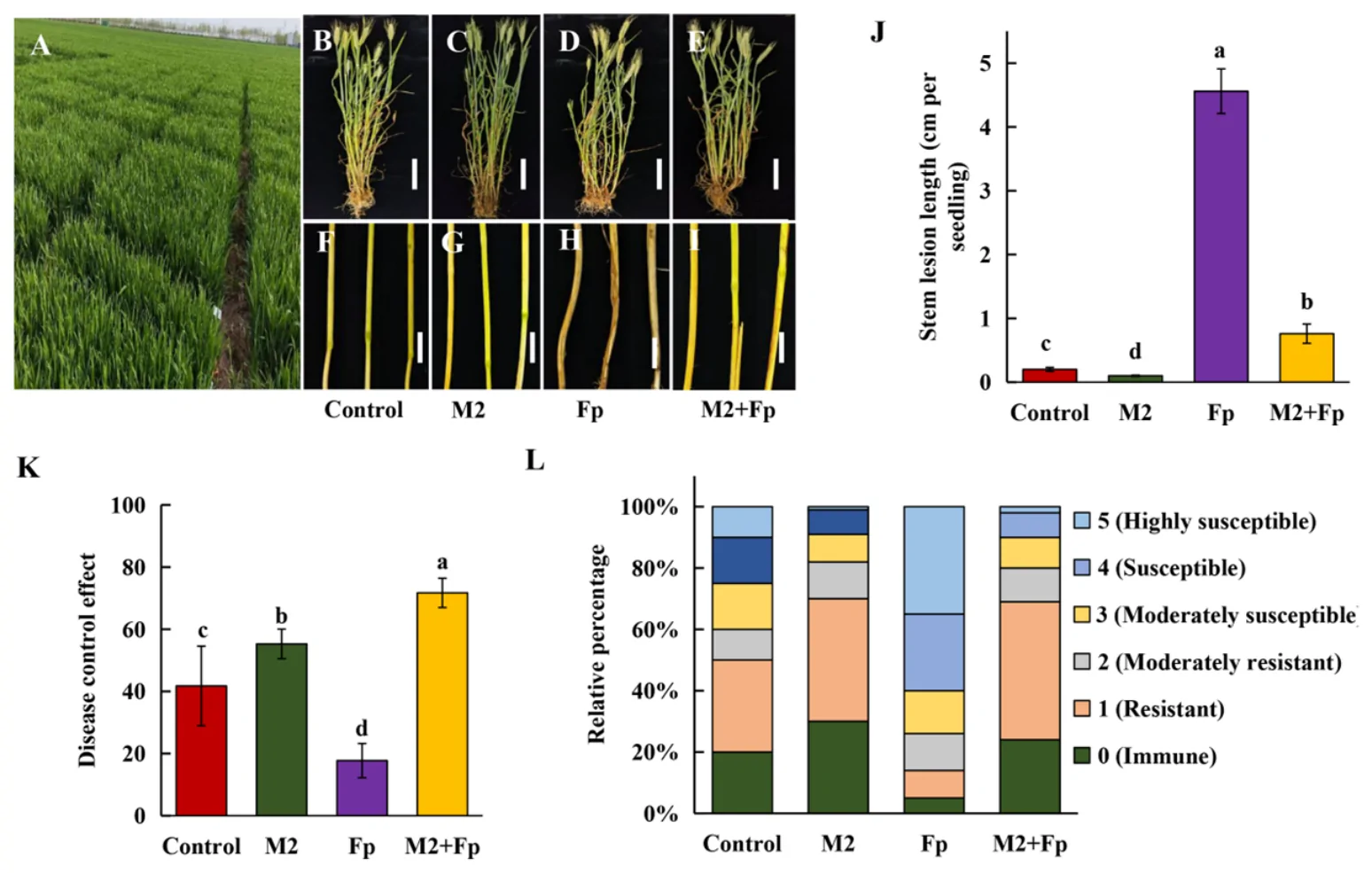
Disease resistance test of the M2 strain under different treatments in the field. (**A**) Plot layout in the field; (**B**–**E**) individual wheat phenotypes (n = 20, respectively); (**F**–**I**) wheat main stem phenotype (n = 20, respectively); (**J**) stem lesion length of wheat (n = 20, respectively); (**K**) disease control effect (n = 100, respectively); (**L**) the number of plants counted under different susceptibility classes (n = 200, respectively). Scale bars: 10 cm in (**B**–**I**). All experiments were repeated three times. Error bars represent the SE of means. Different letters at the top of the bars indicate significance (*p* < 0.05, one-way ANOVA followed by Tukey’s multiple comparison test, n = 10).

**Figure 6:**
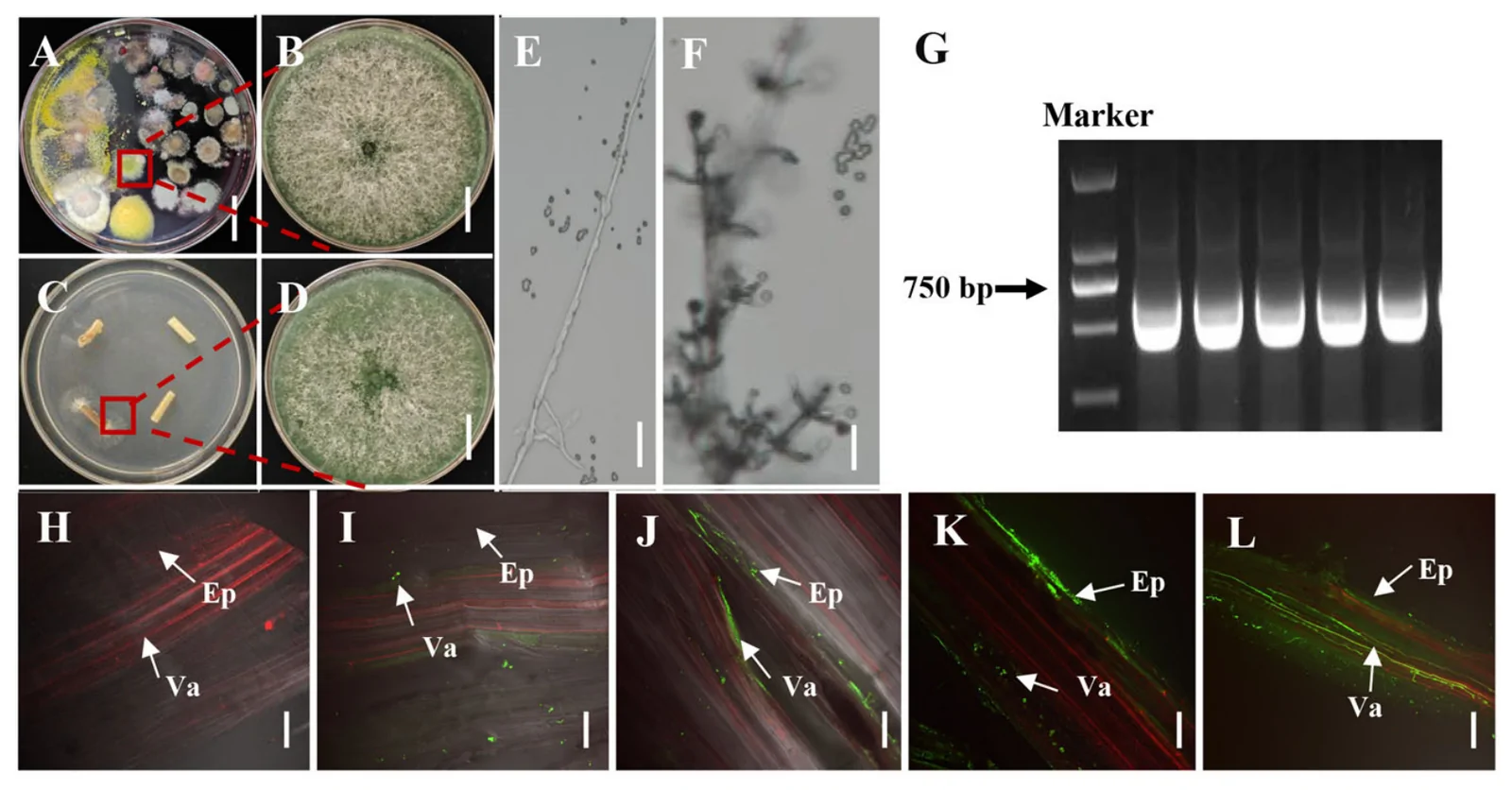
Observation of the colonization of M2. (**A**) Re-separation of M2 in the rhizosphere soil of wheat through Bengali red medium; (**B**) morphological observation of *Trichoderma* at the 5th day; (**C**) re-separation of M2 in the wheat roots and stem bases using the PDA medium; (**D**) morphological observation of *Trichoderma* at the 6th day; (**E**,**F**) morphological observation of M2 hyphae, conidia and conidiophores; (**G**) PCR detection of GFP fragments in wheat tissues; (**H**–**L**) the colonization of *Trichoderma* in the roots of wheat was observed through WGA-AF488 every two days. Ep, epidermis; Va, vascular bundle. Scale bars: 2 cm in (**A**–**D**); 20 μm in (**E**,**F**); 50 μm in (**H**–**L**).

**Figure 7:**
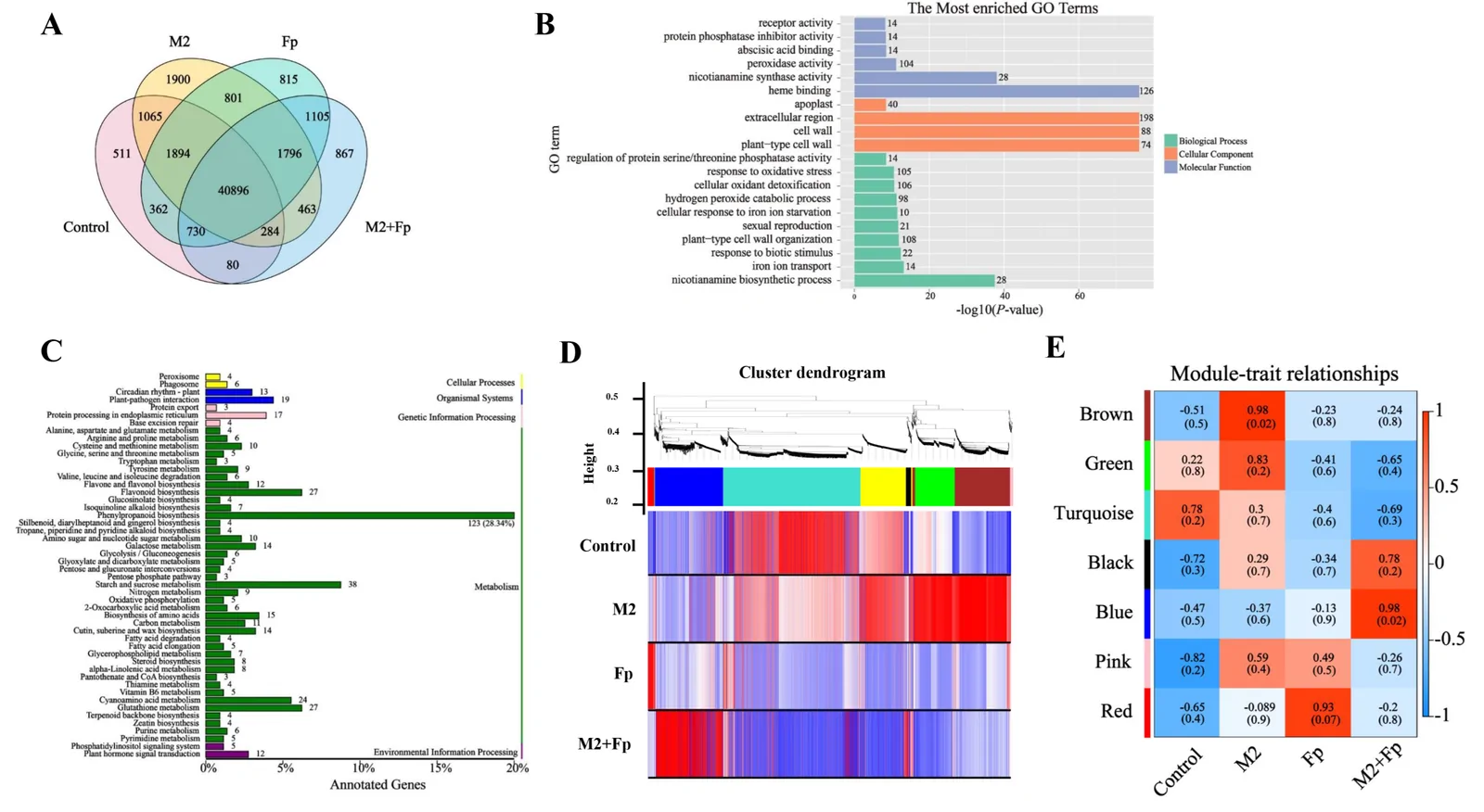
RNA-seq of wheat resistance induced by M2. (**A**) Statistical analysis of DEGqs in sequenced samples; (**B**) GO analysis of differentially expressed genes in M2 treatment; (**C**) KEGG analysis of differentially expressed genes in M2 treatment; (**D**) gene cluster analysis based on WGCNA; (**E**) correlation analysis between gene modules and traits.

**Figure 8:**
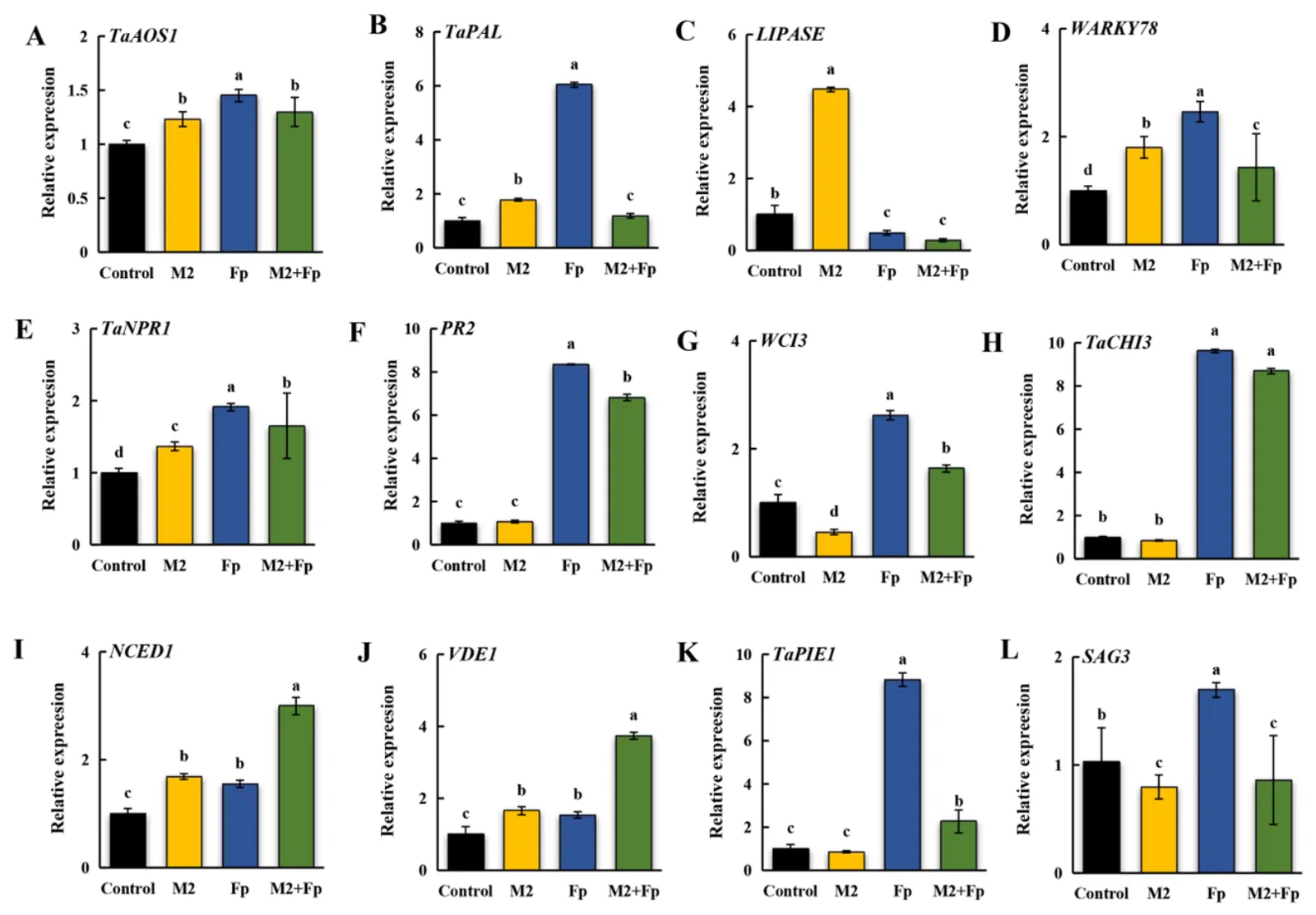
General defense response genes’ expression levels 48 h after inoculation. Different letters indicate significant difference (*p* < 0.05, one-way ANOVA followed by Tukey’s multiple comparison test). (**A**–**D**) *TaAOS1*, *TaPAL*, *LIPASE*, and *WRKY78* are genes associated with JA synthesis in plants [24]; (**E**,**F**) *TaNPR1* and *PR2* are genes associated with SA synthesis in plants [25]; (**G**,**H**) *WCI3* and *TaCHI1* are genes involved in general defense response in plants [27]; (**I**,**J**) *NCED1* and *VDE1* are genes associated with ABA synthesis in plants [27]; (**K**) *TaPIE1* is a gene associated with ethylene synthesis in plants [26]; (**L**) *SAG3* is a senescence marker gene in wheat plants [27]. All experiments were repeated three times, with three independent biological replicates. Error bars represent the SE of means. Different letters at the top of the bars indicate significance (*p* < 0.05, one-way ANOVA followed by Tukey’s multiple comparison test, n = 3).

**Figure 9:**
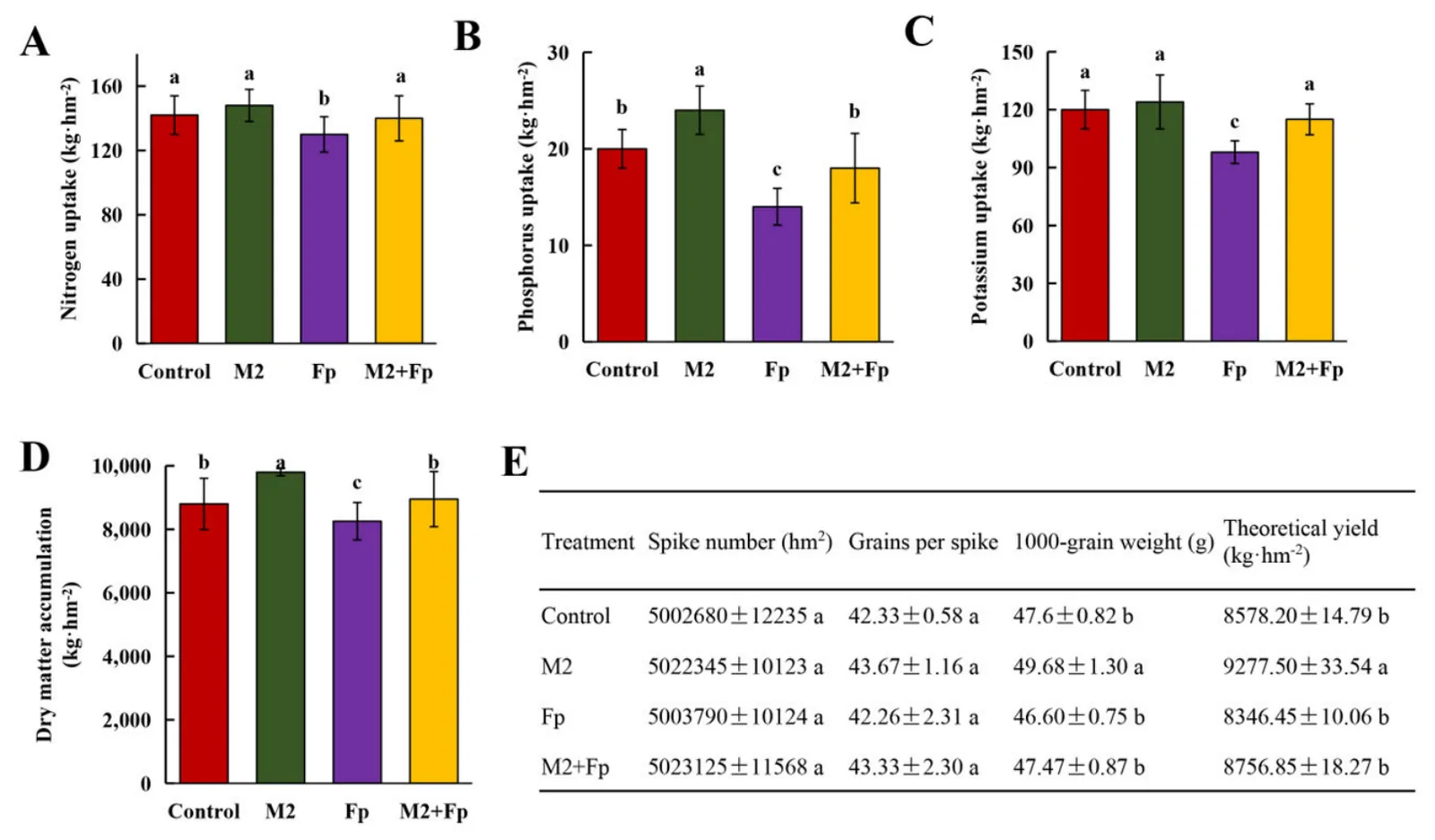
Effect of different treatments on nutrient uptake and wheat yield. (**A**) Nitrogen uptake in different treatments; (**B**) phosphorus uptake in different treatments; (**C**) potassium uptake in different treatments; (**D**) dry matter accumulation in different treatments; (**E**) effects of different treatments on wheat yield components. Error bars represent the SE of means. All experiments were repeated three times, with three replicates. Different letters at the top of the bars indicate significance (*p* < 0.05, one-way ANOVA followed by Tukey’s multiple comparison test, n = 3).

## Data Availability

*T. harzianum* M2 was stored in the China General Microbiological Culture Collection Center. The original number is T-M2, and the preservation number is CGMCC NO. 41972 (https://cgmcc.net/ (accessed on 21 May 2026)). The corresponding accession numbers of the ITS and RPB2 loci of M2 in the NCBI (https://www.ncbi.nlm.nih.gov/ (accessed on 21 May 2026)). Raw transcriptomic data of RNA-seq can be accessed on the website of the National Center for Biotechnology Information (https://www.ncbi.nlm.nih.gov/ (accessed on 21 May 2026)), and the BioProject number is PRJNA1247731. The original contributions presented in the study are included in the article/Supplementary Material; further inquiries can be directed to the corresponding author.

## Supplementary Materials

The following supporting information can be downloaded at: https://www.mdpi.com/article/10.3390/plants15192957/s1, Figure S1. The characteristics of the colony, hypha, conidiophore and conidia of strain M2; Figure S2. Scanning images of wheat roots at different M2 spore concentrations; Figure S3. Morphological diagram of wheat seedlings in the in vitro RNA-Seq experiment (n = 20); Figure S4. PCA analysis among 12 sequencing samples in RNA-seq; Figure S5. PCC analysis among 12 sequencing samples in RNA-seq; Figure S6. The most enriched DEGs between M2+*Fp* treatment and control; Figure S7. The main KEGG pathway of DEGs between M2+*Fp* treatment and control; Table S1. Information on the gene sequences used for phylogenetic analyses; Table S2. The influence of M2 in wheat agronomic traits; Table S3. The influence of M2 in wheat root; Table S4. Disease susceptibility of wheat FCR in the glasshouse; Table S5. Disease susceptibility of wheat FCR in the field; Table S6. Enriched GO terms in the brown and blue modules via WGCNA; Table S7. Enriched KEGG pathways in the brown and blue modules; Table S8. DNA sequences of the primers used in this study.

## Abbreviations

The following abbreviations are used in this manuscript:

FCR	Fusarium crown rot
DI	disease index
ET	ethylene
*Fc*	*F. culmorum*
*Fg*	*F. graminearum*
*Fm*	*F. moniliforme*
*Fo*	*F. oxysporum*
*Fp*	*F. pseudograminearum*
FDR	false discovery rate
FC	fold change
GFP	green fluorescent protein
ISR	induced systemic resistance
JA	jasmonic acid
PR	pathogen-related
PCC	Pearson correlation coefficient
PDA	potato dextrose agar
PCA	principal component analysis
RDI	relative disease index
SA	salicylic acid
SAR	systemic acquired resistance
