# Supplementary material for: Trichoderma harzianum M2 Induces Systemic Resistance to Fusarium Crown Rot and Remodels Root Architecture to Promote Phosphorus Uptake in Wheat
Source: Plants (Basel). 2026 Sep 28;15(19):2957. doi: 10.3390/plants15192957 (PMC13667173; doi:10.3390/plants15192957)
Supplement: Supplementary file 1 [file plants-15-02957-s001.zip › Supplementary Figures.pdf]

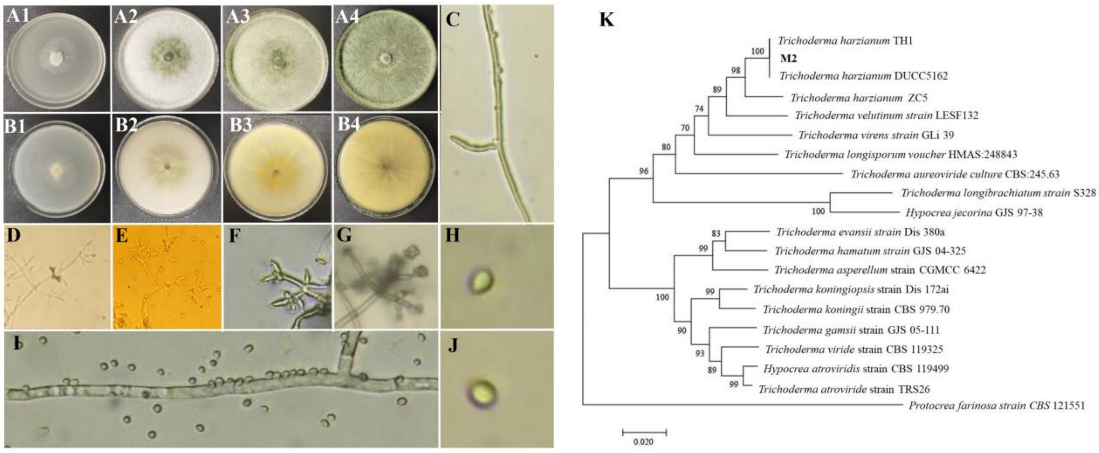

Figure S1 The characteristics of the colony, hypha, conidiophore and conidia of strain M2
(**A-B**) Morphological observation of strain M2 every two days, the front of strain M2 in group A (**A1-A4**) and the back of strain M2 in group B (**B1-B4**); (**D-E**) are conidial peduncules of M2; (**F-G**) are the stalks of M2; (**C, I**) are mycelium; (**H, J**) are conidia. (**K**), phylogenetic tree analysis of M2

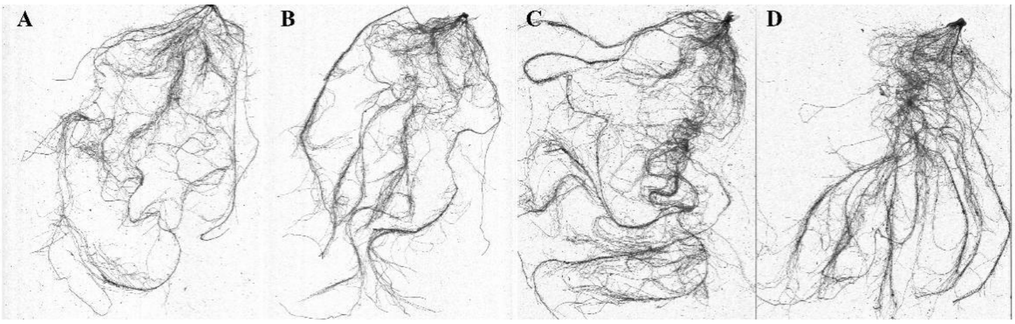

Figure S2 Scanning images of wheat roots at different M2 spore concentrations
(**A**) is Control; (**B**) is the root morphology of wheat with M2 spore concentration under 10^3 cfu/mL; (**C**) is the root morphology of wheat with M2 spore concentration under 10^5 cfu/mL; (**D**) is the root morphology of wheat with M2 spore concentration under 10^7 cfu/mL

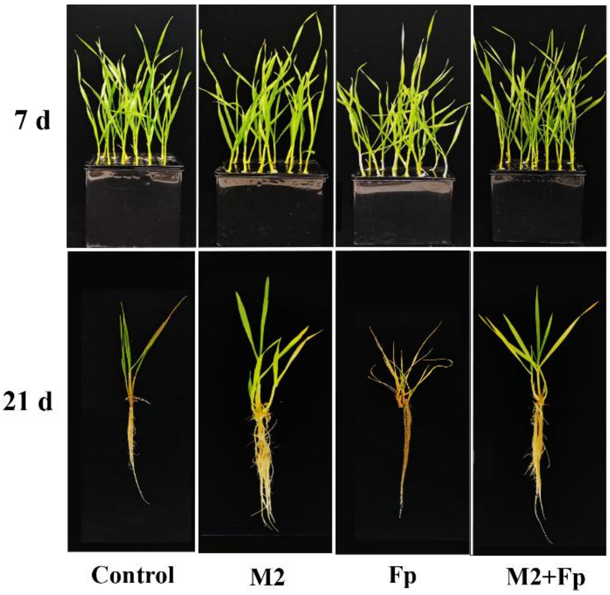

Figure S3 Morphological diagram of wheat seedlings in the *in vitro* RNA-Seq experiment (n=20).

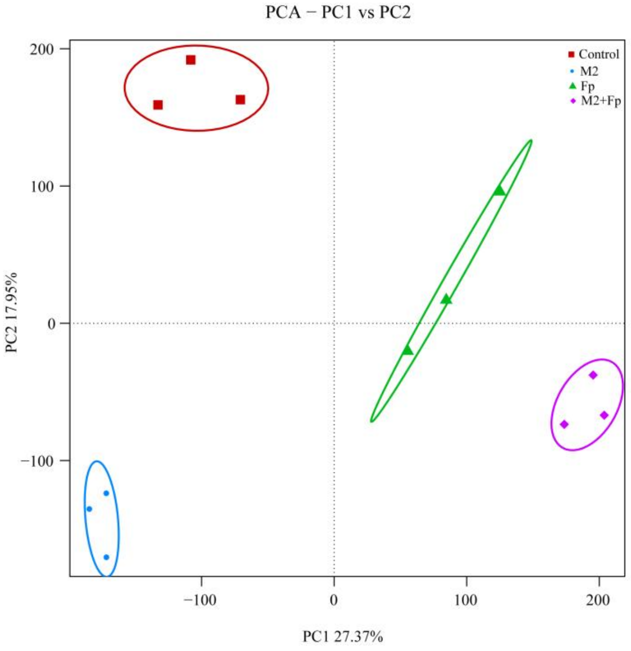

Figure S4 PCA analysis among 12 sequencing samples in RNA-seq

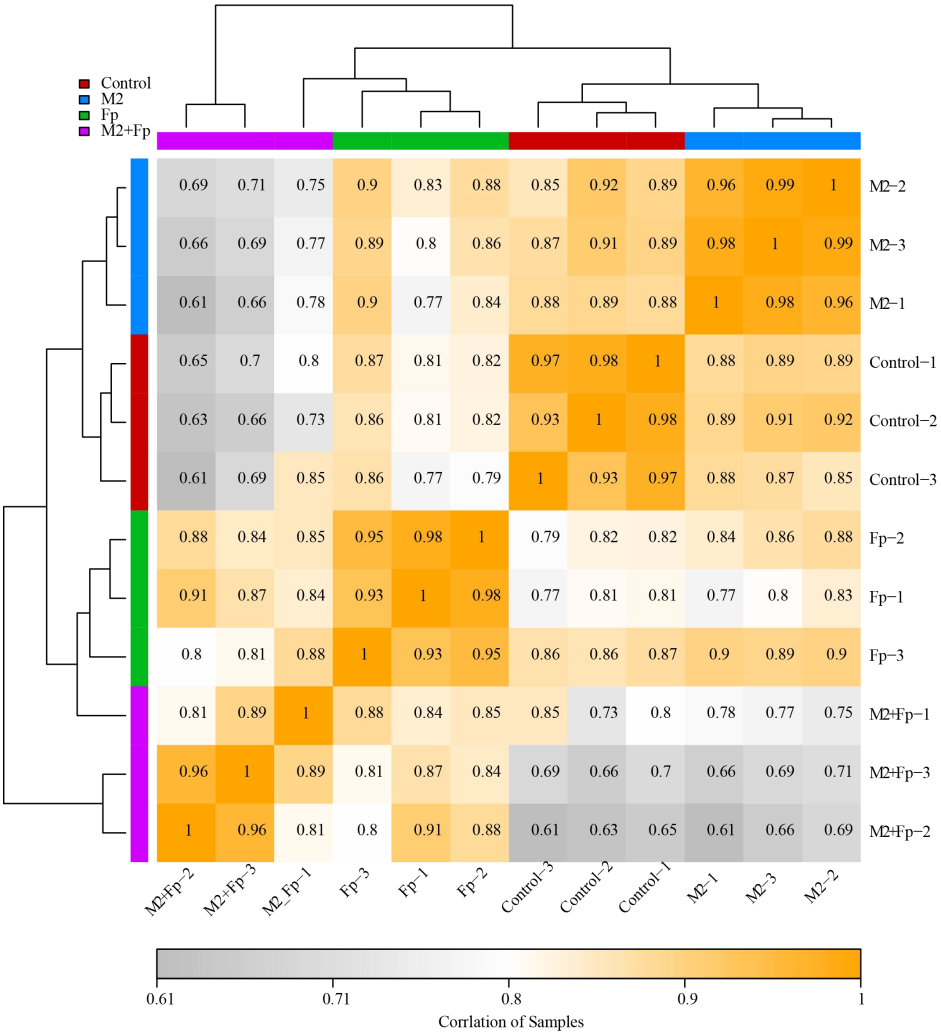

Figure S5 PCC analysis among 12 sequencing samples in RNA-seq

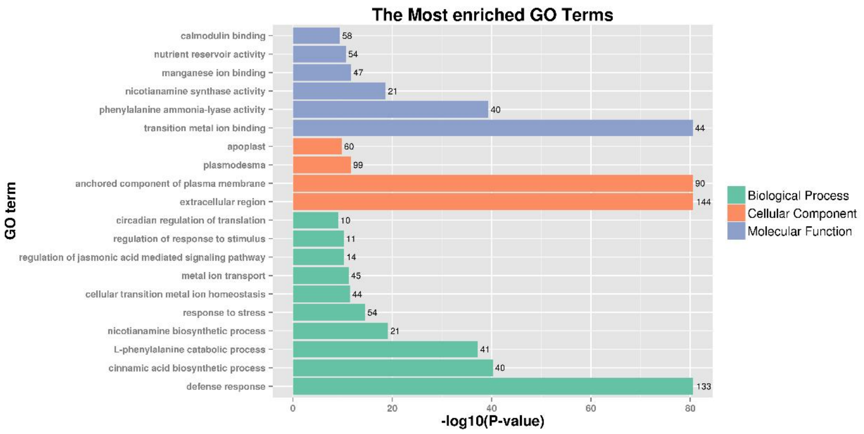

Figure S6 The most enriched of DEGs between M2+*Fp* treatment and Control

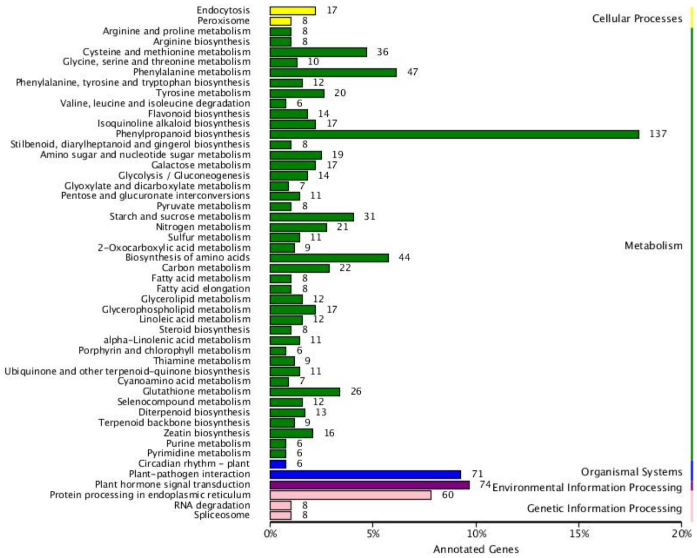

Figure S7 The main KEGG pathway of DEGs between M2+*Fp* treatment and Control
